# Hardware and Software Setup for Quantitative ^23^Na Magnetic Resonance Imaging at 3T: A Phantom Study

**DOI:** 10.3390/s24092716

**Published:** 2024-04-24

**Authors:** Giulio Giovannetti, Alessandra Flori, Nicola Martini, Filippo Cademartiri, Giovanni Donato Aquaro, Alessandro Pingitore, Francesca Frijia

**Affiliations:** 1 Institute of Clinical Physiology, National Council of Research, Via G. Moruzzi 1, 56124 Pisa, Italy; giulio.giovannetti@cnr.it (G.G.); pingi@ifc.cnr.it (A.P.); 2 Bioengineering Unit, Fondazione G. Monasterio CNR-Regione Toscana, 56124 Pisa, Italy; alessandra.flori@ftgm.it (A.F.);; 3 Department of Radiology, Fondazione G. Monasterio CNR-Regione Toscana, 56124 Pisa, Italy; fcademartiri@ftgm.it; 4 Department of Surgical, Medical, Molecular and Critical Area Pathology, University of Pisa, 56126 Pisa, Italy; giovanni.aquaro@unipi.it

**Keywords:** ^23^Na magnetic resonance imaging, ^23^Na coil design

## Abstract

Magnetic resonance (MR) with sodium (^23^Na) is a noninvasive tool providing quantitative biochemical information regarding physiology, cellular metabolism, and viability, with the potential to extend MR beyond anatomical proton imaging. However, when using clinical scanners, the low detectable ^23^Na signal and the low ^23^Na gyromagnetic ratio require the design of dedicated radiofrequency (RF) coils tuned to the ^23^Na Larmor frequency and sequences, as well as the development of dedicated phantoms for testing the image quality, and an MR scanner with multinuclear spectroscopy (MNS) capabilities. In this work, we propose a hardware and software setup for evaluating the potential of ^23^Na magnetic resonance imaging (MRI) with a clinical scanner. In particular, the reliability of the proposed setup and the reproducibility of the measurements were verified by multiple acquisitions from a 3T MR scanner using a homebuilt RF volume coil and a dedicated sequence for the imaging of a phantom specifically designed for evaluating the accuracy of the technique. The final goal of this study is to propose a setup for standardizing clinical and research ^23^Na MRI protocols.

## 1. Introduction

Although the detection and the quantification of the total sodium (^23^Na) in biological tissues were initially investigated using MR spectroscopy in the early 1970s and using magnetic resonance imaging (MRI) in the early 1980s [1,2,3,4,5,6,7], in recent years, the scientific interest in sodium MRI has experienced a significant increase due to its physiological and pathophysiological relevance. In particular, sodium significantly contributes to the potential maintenance of the resting cell membrane, mainly through the action of the Na^+^/K^+^-ATPase pump [8], while its accumulation has been measured under different pathologic conditions such as tumors [9], ischemia [10], and neurodegenerative diseases, including multiple sclerosis [11] or Alzheimer’s disease [12].

The use of ^23^Na MRI offers the possibility of extending anatomical imaging by providing additional and complementary information regarding both physiology and cellular metabolism.

In particular, ^23^Na MRI allows for the non-invasive quantification of ^23^Na concentration in the target tissue through the assessment of the so-called tissue sodium concentration (TSC), which can be considered a biomarker of cell viability and integrity. This could provide additional information regarding the entity and severity of damaged tissue, as well as the probability of functional recovery. Sodium concentration is, in fact, strictly dependent on the tissue metabolic state and on the integrity of the cell membrane [13]. Pathologies for which cell membrane integrity is compromised can lead to an increase of the tissue sodium concentration. Increased TSC levels have been detected in ischemic stroke lesions [10,14] and in myocardial infarction [15,16].

For instance, in acute myocardial infarction, a peak of TSC has been found within the first day, which reduces progressively in the following days due to the healing process, while in patients with chronic myocardial infarction, a myocardial TSC increase has been documented, without any correlation with infarct size, left ventricular function, or the occurrence of arrhythmias [17].

In the context of cardiovascular prevention, ^23^Na MRI has been performed in patients with diabetes and hypertension, measuring skin and muscle TSC. A good reliability and reproducibility regarding the measurement of the sodium concentration in skin and muscle was found in the study in Ref. [18].

Thus, these data are of interests, showing the potential of ^23^Na MRI to provide additional information regarding the entity and severity of damaged tissue.

Moreover, the capacity of ^23^Na MRI to measure cell density can provide important information regarding the efficacy of chemotherapy in patients with cancer. This ability has been mainly studied in patients with brain cancer, showing the potential of ^23^Na MRI to guide a personalized and flexible treatment, rather than relying on a rigid therapeutic protocol based on the response of the tumor mass to therapy [19]. Other studies have been performed for breast and prostate cancer [9].

The ^23^Na MRI method allows for the tracking of pathologic changes in skeletal muscle tissue, in the kidney, and in articular cartilage [20], and it has been explored as a non-invasive tool to investigate tissue abnormalities in patients with multiple sclerosis [11] and Alzheimer’s disease [21].

Due to the low biological concentration and the low gyromagnetic ratio (γ_Na_ = 11.26 MHz/T, approximately one-quarter that of hydrogen at γ_H_ = 42.57 MHz/T), achieving an acceptable signal-to-noise ratio (SNR) is challenging in sodium MRI studies. The lower sodium Larmor frequency requires custom radiofrequency (RF) coils, and the MR scanner must include multinuclear spectroscopy (MNS) capabilities.

In general, all RF coils designed to be employed in an MR scanner must satisfy compatibility and safety criteria. In particular, homebuilt RF coils must be compatible with commercial scanners. In this case, the interconnection to the scanner is achieved via 50 Ω coaxial cables, since their dielectric material quality and shield design allow for the RF energy transport as low-loss lines. Indeed, cables that are not properly terminated in their characteristic impedance may be subjected to standing waves and common mode currents, which could heat the patient/sample and irradiate energy in the environment. Since different MR scanners employ various and proprietary connectors, a strong collaboration between researchers and scanner manufacturers is a constraint when designing homebuilt RF coils. Coils must be matched to 50 Ω in order to optimize the energy transfer through all parts of the spectrometer, ensuring that the system impedance exhibits a pure 50 Ω resistance. To achieve this, different impedance matching circuits can be specifically designed and built, generally comprised of inductors and/or capacitors [22]. 

For coils designed in the transmitter/receiver mode with clinical scanners, a T/R switch provided by the scanner manufacturer must be inserted between the coil and the scanner, which also has the function of monitoring the specific absorption rate (SAR) for quantifying the power deposited on a subject to avoid dangerous tissue heating [23].

When using quadrature coils, a −3 dB coupler circuit must be employed for equally splitting the transmitting power into two channels, while introducing a 90° phase shift in one of the channels. These two channels are then fed to produce two orthogonal magnetic fields. The same -3dB coupler circuit must be used to combine the two signals, with a 90° phase correction during the reception phase [24]. Finally, fixed and variable capacitors for coil tuning and matching are specially designed by many manufacturers for MR applications in a high-quality factor (Q) non-magnetic version and with high breakdown voltage, while the inductors can be easily homebuilt. 

As a further issue, due to the low in vivo concentration and small nuclear magnetic resonance sensitivity of sodium, ^23^Na MR imaging usually requires long acquisition times when compared with ^1^H MRI. Furthermore, the sodium nucleus possesses very short relaxation times (T1 is of the order of a few tens of ms, while T2 is characterized by a bi-exponential decay, with a fast component of about 2–4 ms and a slower component of about 12–40 ms, depending on the specific anatomical district [25,26]), which necessitates the use of dedicated ultrashort echo time (UTE) sequences to enable quantitative measurements.

With the increasing magnetic field strength of MRI scanners, the improved hardware capabilities, such as strong gradient strengths with high slew rates, and new dedicated radiofrequency ^23^Na coils, it is now possible to reach reasonable measurement times (~10–15 min) with a resolution of a few millimeters [27]. 

For example, by using a homemade dual-tuned ^1^H/^23^Na volume coil [28], Giovannetti et al. performed ^23^Na chemical shift imaging (CSI) of a human calf from a healthy volunteer using a 3T scanner, including multi-nuclear spectroscopy (MNS) capabilities, obtaining good quality ^23^Na maps. Another in vivo experiment carried out on a second volunteer using a reference ^23^Na phantom (38 mM NaCl) permitted the production of quantitative ^23^Na CSI maps: the measured ^23^Na concentration resulted in the range of 15–30 mM, in agreement with literature data for muscle sodium concentration [26].

However, as for quantitative MRI, repeatability, reproducibility, and accuracy measurements are necessary in order to assess the reliability of the technology and to support the relevance of TSC as an in vivo biomarker. This assessment would aid in the understanding of longitudinal data, as well as in regards to multi-scanner data comparison. 

The goal of this study was to test the reproducibility of ^23^Na MRI acquisitions on a 3T scanner, as well as the medium-term (ranged from 1–40 days) repeatability of sodium quantification [29]. 

We reported the measurements of the sensitivity of a homebuilt volume coil employed for the 3T scanner experiments by using the perturbing sphere method [30,31], useful for fast periodic coil quality controls. Finally, we obtained and compared sodium concentration maps of a dedicated homemade sodium phantom, scanned three times during several weeks to mimic the variability of the proposed approach.

## 2. Materials and Methods

### 2.1. Coil Sensitivity Measurement

**Figure 1:**
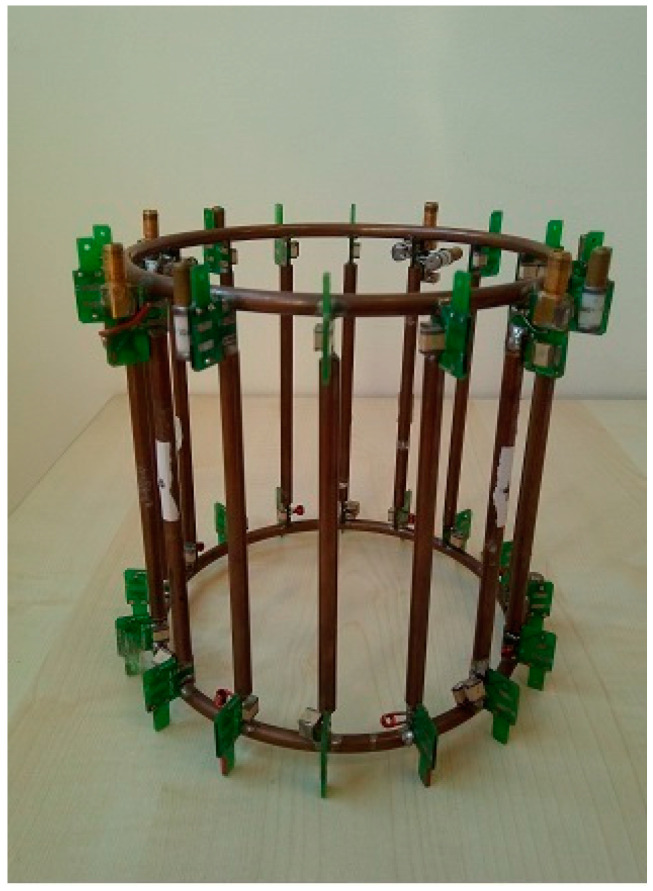
The homebuilt dual-tuned ^1^H/^23^Na coil.

The coil was used on a 3T whole-body MRI scanner (HDx TWINSPEE, GE Healthcare, Waukesha, WI, USA). It was a dual-tuned ^1^H/^23^Na volume coil, consisting of a 15 cm length–15 cm diameter lowpass birdcage, with alternate tuning of the legs and trap circuits for decoupling the H and Na channels. This coil prototype (Figure 1) was previously employed for acquiring data from phantom and in vivo acquisitions on human calves using a 3T MRI scanner [28].

In this paper, we further characterized the coil employed for the 3T acquisitions by measuring its sensitivity, which is another important parameter that illustrates the RF coil performance. Coil sensitivity is defined as the magnetic field (*B*_1_) induced by the RF coil at a given point per unit of supplied power *P*, as follows [32]:

(1) $$\eta =\frac{B_{1}}{\sqrt{P}}$$

The reciprocity theorem [33] allows for the use of the same quantity defined in Equation (1) to characterize both the transmit and receive performance of an RF probe. It is important to note that maximizing the coil sensitivity will also maximize the SNR [34].

During the test, the coil sensitivity *η* was measured using the perturbing sphere method, an electromagnetic bench test originally employed for the magnetic field mapping of X-band microwave cavity resonators [30] and more recently applied to map the RF fields from MRI coils, with an accuracy comparable with that provided by the standard methods of calibrating the B_1_ field in MR experiments [35]. This method was even employed for comparing the coil sensitivities, measured inside and outside of the scanner, in order to verify whether the eventual increase in coil losses inside the magnet can be ascribed to the position of the coil inside the magnet, which can couple with the system body coil [31].

The perturbing sphere method consists of putting a small metallic sphere inside the cavity of the coil and measuring the shifted frequency *f*_1_ with respect to the unloaded coil. Then, the following equation is used:

(2) $$\eta =\frac{B_{1}}{\sqrt{P}}=\frac{1}{2}\sqrt{\left(\frac{\mu_{0}}{\pi^{2}B_{w}{r_{S}}^{3}}\right)\left(\frac{{f_{1}}^{2}-{f_{0}}^{2}}{{f_{0}}^{2}}\right)}$$

where *B*_1_ is the rotating component of the magnetic field for a linearly polarized coil, *B_w_* and *f*_0_ are, respectively, the −3 dB bandwidth and the coil resonant frequency, and *r_s_* is the sphere radius.

For circularly polarized coils, such as the birdcage used in this work, the terms 1/2 has to be substituted with $\frac{1}{\sqrt{2}}$, since the power will be split to drive the two quadrature channels.

Equation (2) is valid when the sphere is placed in a region of a zero electric field, such as at the center of the birdcage coil volume cavity, where the electric field is negligible [24].

For frequency and bandwidth measurements, a homebuilt dual-loop, consisting of two pickup loops, and an HP3577 network analyzer (Hewlett Packard, Palo Alto, CA, USA) were employed. The input ports of the coil were open, which means the measured *B_w_* is multiplied by a factor two. The network analyzer was set in averaging mode to improve measurement sensitivity.

It is important to underline that the perturbing sphere method is sensitive to both electrical and magnetic field components; therefore, it allows for the measurement of coil sensitivity with great accuracy, only if the electrical and magnetic field components are well separated in space or in regions of a zero electric field. When a separation of the two component contributions is required (i.e., for very high frequency-tuned coils), the sphere must be replaced with conductors of different shapes [31].

### 2.2. MRI Acquisition Protocol and Postprocessing of the ^23^Na Sequence

**Figure 2:**
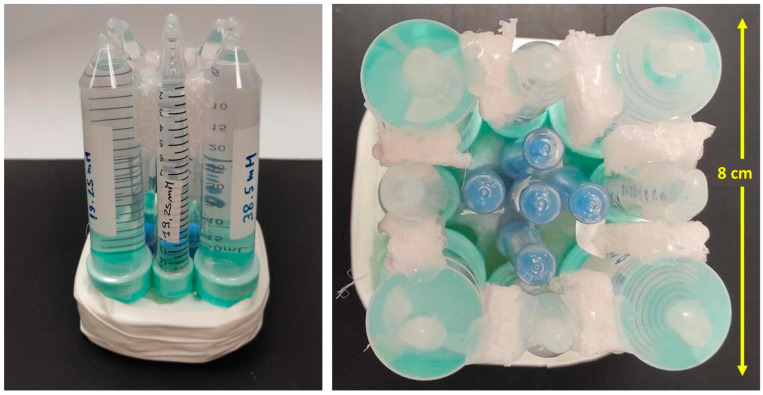
The homebuilt phantom used for testing.

A dedicated phantom has been built in order to investigate the performance of the experimental setup (coil and sequence) in terms of both sensitivity (in discriminating different ^23^Na concentrations) and spatial resolution. The phantom is constituted of plastic vials of three sizes (large: 50 mL volume, medium: 15 mL, and small: 7 mL), each filled with NaCl salt solutions at four different concentrations: 9.625, 19.25, 38.5, and 77 mM (Figure 2). The different ^23^Na concentrations were obtained using sequential water dilutions of saline (Baxter). The central vial (7 mL) was filled with pure saline for reference (154 mM). The vials were positioned roughly in a circle in order to produce a symmetric geometry.

Acquisitions were performed using imaging sequences from the Multinuclear Spectroscopy (MNS) package (GE Healthcare, Munich, Germany), developed and optimized for multinuclear studies. The calibration of transmit gain (TG) and the right frequency were set using the Bloch–Siegert pulse [36]: after the excitation pulse, the Bloch–Siegert pulse induces a transmit field (*B*_1_^+^)-dependent phase shift. After the slice-refocusing gradient, the acquisition begins. At least two acquisitions, with plus and minus the off-resonance frequency, are required.

A flip angle (FA) calibration was first performed to select the optimal FA leading to the higher SNR: five measurements were carried out, with a linearly nominal flip angle increasing from 10° to 50°, and the best SNR value, measured as the average signal intensity in the large cylindrical vial at 77 mM, divided by the standard deviation of the noise, was evaluated for each acquisition [37,38]. 

Data were acquired with a 3D radial trajectory with a golden-angle rotation, using the following parameters: hard pulse excitation; FOV = 38.4 cm; nominal resolution 1.86 × 1.86 mm; radial spokes 15,460; TR = 5 ms; TE = 0.5 ms; FA = 30°; slab = 20 cm, leading to an overall scan time of 1:17 min. The sequence was repeated 12 times, and the raw data were averaged to improve the SNR. Parameters for the radial readout were a readout duration of 5 ms and a maximum gradient strength of 33 mT/m.

The examinations were repeated three times within a single MRI session (intra-day) and every week for three weeks (inter-day) to assess the variability of the measurements over a medium-term period. 

The radial raw datasets were reconstructed offline using Matlab R2020b (The MathWorks, Inc., Natick, MA, USA). Reconstruction consisted of a non-uniform Fourier transform (NUFFT) [39] of the acquired k-space data, with a reconstructed resolution of 1.5 mm.

All the images were aligned using the ITK-SNAP 4.0.2 tool [40], initially employing a manual alignment, then using an automatic alignment, with the cross correlation with the similarity metric method.

**Figure 3:**
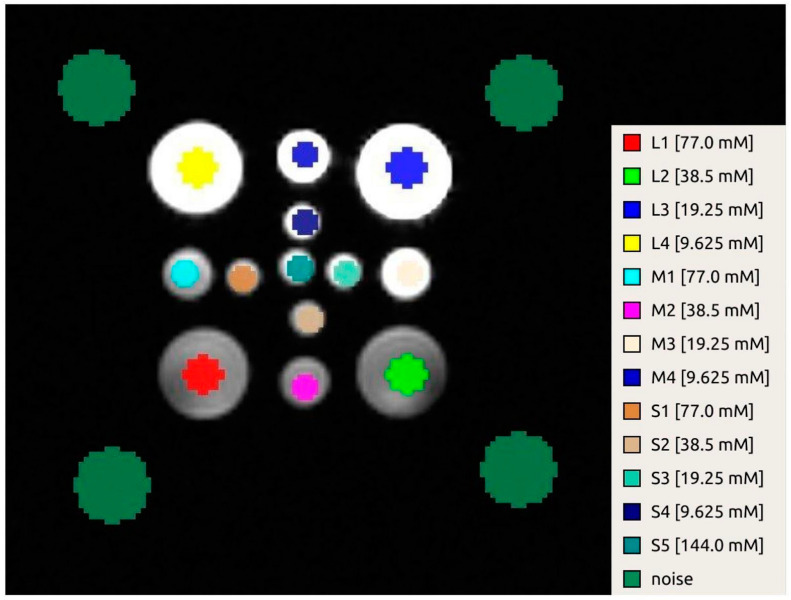
Localization of the ROIs on a reference ^1^H image.

Once the datasets were spatially aligned, 13 circular ROIs were drawn in the central axial slice, each placed in the center of the vial (Figure 3). The diameter of the ROIs was 12 mm for the large vials and 8 mm for the others. The noise distribution was estimated from four circular ROIs (diameter of 22.5 mm), placed in the background around the corners of the phantom.

Sodium quantitation was then performed using linear regression in Matlab. ROIs were drawn in the four large vials on the phantoms (9.625, 19.25, 38.5, and 77 mM NaCl), and their average signal intensities were measured. Another ROI was drawn in the noise background, and the mean value of the noise was used as a 0 mM sodium concentration phantom. A linear regression curve of these phantom intensities and noise versus sodium concentrations was then calculated and used to extrapolate the sodium concentration maps of all the vials in the phantom [41].

The repeatability and reproducibility of the measurements of ^23^Na concentration were evaluated through the coefficient of variation (CoV), defined as the percentage ratio between the standard deviation and the mean of the ^23^Na concentration values obtained over repeated measurements [42]. Intra-day repeatability was calculated as the CoV for repeated measurements in the same experimental session (n = 3), while inter-day repeatability was evaluated by calculating the CoV of the first acquisition over different weekly imaging sessions (n = 3).

## 3. Results

### 3.1. Coil Sensitivity Measurement

The coil was tested using a 11.5 mm radius steel sphere for both ^1^H and ^23^Na frequencies. The coil sensitivity values, calculated using Equation (2) as the average of four measurements, with a standard deviation, are 12.76 ± 0.99 and 1.30 ± 0.06, respectively, at 33.78 MHz (^23^Na @3T) and 127.75 MHz (^1^H @3T) frequencies.

The difference in performance at the two frequencies is due to the lower conductor resistance and higher capacitor quality factor provided at the lower frequency, which was confirmed by the unloaded quality factor measurements (291 and 147 for the ^23^Na and ^1^H frequencies, respectively). Moreover, a further loss of efficiency, measured at proton frequency, depends on the losses in the traps, which was confirmed by the coil simulation results [28], since the magnetic field intensity at the sodium frequency was greater than the one produced at the proton frequency, by a value greater than 5 dB.

However, as described in Ref. [28], both magnetic fields at proton and sodium frequencies were homogeneous, reproducing the typical magnetic field pattern of a birdcage coil and denoting the high decoupling between the proton and sodium channels.

These results demonstrate that the dual-tuned coil configuration permitted high performance, providing anatomical localization and sodium data collection in sequence, without repositioning the sample.

### 3.2. Repeatability Analysis of Sodium Measurement Concentration

**Figure 4:**
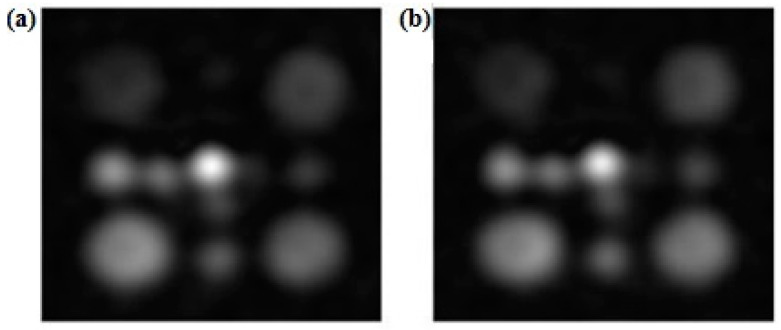
Comparison of ^23^Na images obtained at different flip angles: (**a**) FA = 15°; (**b**) FA = 30°.

Figure 4 shows representative ^23^Na images obtained with the two different FAs: 15° and 30° (Figure 4a and Figure 4b, respectively). The FA calibration indicated that the optimal FA for ^23^Na MRI under the experimental conditions proposed in this work was 30°.

As can be observed from Figure 4, the large vials (50 mL) could be clearly detected, regardless of their concentration value. The medium vials could be clearly identified for the 19.25, 38.5, and 77 mM concentrations, while only the small vials are distinguishable in the ^23^Na images at higher concentration (38.5 and 77 mM). From the images at FA = 15° and FA = 30°, we measured the SNR in the large cylindrical vial at 77 mM; the SNR is higher for the FA at 30° for all three days (Table 1); therefore, for the image acquisition, we chose FA = 30°. 

**Figure 5:**
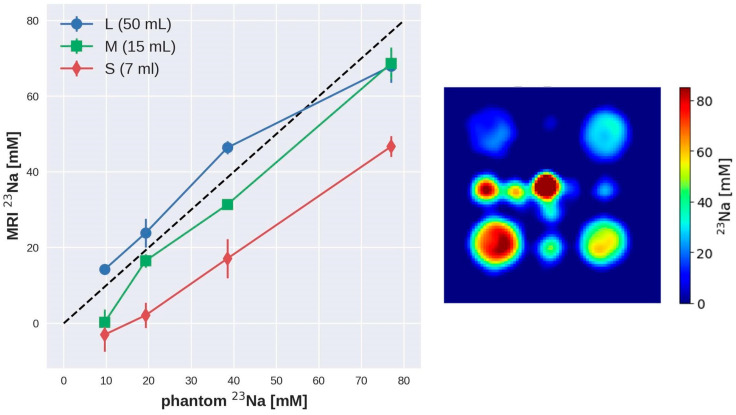
The ^23^Na concentrations estimated from the sodium images obtained at 3T in the phantom, with the optimized flip angles of 30°, are reported as a function of the actual concentration. The mean values calculated across inter-day experiments are reported in the graph; the error bars indicate the standard deviation. The dashed line represents the identity line (Y = X). The ^23^Na concentration map was acquired with the optimized flip angles of 30°.

Figure 5 shows the relationship between the actual ^23^Na concentration values in the phantom vials and those estimated from the ^23^Na MRI maps obtained across inter-day experiments using the proposed experimental setup. A linear relationship was found for the ^23^Na concentrations in all the vials, with the large vials (50 mL) matching the actual concentration. Smaller vials exhibited an underestimation of the concentration that we ascribe to the SNR and spatial resolution of the ^23^Na MR images, which are inadequate for these vials.

To evaluate the performance and the reliability of the hardware and software setup proposed in this work, repeatability and reproducibility measurements were conducted in the phantom at 3T over a medium time interval. The mean ^23^Na concentration values estimated across the different MRI sessions (n = 5) for the different vials are reported in Table 2 (mean ± standard deviation). As also shown in Figure 5 for the inter-day tests, the concentration values estimated with the proposed setup across all the MRI sessions performed in this study show a similar trend to that of the theoretical values. However, except for the large vials, the concentrations are generally underestimated as compared to the nominal values.

As reported in Table 3, the CoVs associated to the ^23^Na concentration values obtained among different scans were determined and compared to assess the intra-day (between scans executed over the same day) and the inter-day (between the first scan performed over different days using the same experimental setup) repeatability of the measurements.

## 4. Discussion

The ^1^H -MRI is a non-invasive medical imaging technique that has increasingly become a valuable and widespread diagnostic tool for daily clinical practice. 

MRI is characterized by excellent soft tissue contrast, allowing for the imaging of human anatomy, with high spatial and temporal resolution in essentially all anatomical districts/organs under different pathophysiological conditions.

In parallel, multiparametric quantitative MRI approaches allow for the estimation and mapping of imaging parameters (such as the relaxation times or the apparent diffusion constant), providing semi-quantitative information on tissue pathophysiology.

In the clinical setting, as well as in research applications, the need for quality assurance (QA) or quality control (QC) protocols has become a fundamental requirement for evaluating MRI system performance and ensuring adequate image quality.

QA and QC protocols deal with the establishment of standardized imaging procedures and the identification of test parameters (such as the SNR, the contrast-to-noise ratio (CNR), and the magnetic field homogeneity) and reliable metrics that can provide information on the MRI system performance and the reproducibility and repeatability of the measurements [42]. Moreover, the assessment of repeatability and reproducibility often require the setup of dedicated phantoms [41].

Additionally, huge amounts of data are now made available through the sharing of protocols among different research/clinical centers, which can be used, for instance, for multi-centric studies. Establishing standardized protocols and assessing intra- and inter-scanner reproducibility is thus a prerequisite to ensure reliable data comparison and proper understanding [43].

Because of the reduced intrinsic sensitivity, the development of specific QA protocols becomes still more important for MRI studies performed with heteronuclei, such as 13C or ^23^Na, especially considering the possibility of measuring semi-quantitative parameters such as the TSC [29,37].

The aim of this paper is to provide an experimental setup and methodology to investigate the reproducibility and repeatability of ^23^Na MRI acquisitions over a medium time interval. 

Due to the intrinsic limited sensitivity of the ^23^Na nuclei, the MR hardware employed for acquisition is one of the key components of the experimental setup. To ensure reliable measurements, the performance of the coil must be well characterized and periodically checked. The perturbing sphere method reported in this work has allowed the coil performance to be characterized in a short time and can be useful for periodic coil quality controls.

Some previous papers deal with the repeatability of in vivo TSC determination with ^23^Na MRI [29,37,44]; in our work, we focused on investigating ^23^Na image quality by assessing the intra- and inter-day repeatability of ^23^Na concentration values obtained in a phantom on a 3T scanner. This is propaedeutic to the application of our experimental protocol to human studies. 

In order to investigate the performance of our experimental setup (RF coil and acquisition sequence), we built a dedicated symmetric phantom consisting of plastic vials with different ^23^Na concentration and dimensions. This allowed us to test the accuracy of our system in terms of spatial resolution and at the same time, to investigate the sensitivity of our setup. Looking at the clinical application of ^23^Na MRI, we selected ^23^Na concentration values like those typical of human tissues. Our results obtained in the phantom show that different ^23^Na concentrations in the range of human values could be clearly distinguished with our experimental approach. Some criticality was found for the smaller vials with the lowest concentration (i.e., 9.625 mM), for which we detected a general underestimation of the concentration values (Figure 5 and Table 2). This is due to both the SNR and spatial resolution, which appear to be inadequate to clearly identify these vials in the ^23^Na MR images. The ^23^Na MR acquisition provided a nominal resolution of 1.86 × 1.86 mm (in-plane resolution), while the size of the smaller vials (7 mL volume) was: length = 8.5 cm; outer diameter = 1.2 cm; inner diameter = 1 cm. In this experimental setup, the image quality is heavily determined by the SNR, which was insufficient to image the vials with the lower concentration values. 

The phantom proposed in this work is characterized by a very simple design, easy preparation, and suitability for repeated testing of the performance of the experimental setup. The phantom could be further improved for future testing. For instance, an external container could be added to keep the vials immersed in a liquid, i.e., a ^23^Na solution [45]; this would drastically reduce the presence of air and improve B_0_ homogeneity. Moreover, agarose crystals could be added to the phantom designed for the ^23^Na -MRI studies to better mimic in vivo conditions, especially in terms of ^23^Na relaxation times [37]. In this case, the T2 relaxation process of the ^23^Na solutions follows a bi-exponential behavior, the same behavior that occurs in biological tissues [45]. The use of tissue-mimicking materials, such as agarose, is therefore very helpful for reliable TSC estimation when the phantom is used as a reference in in vivo ^23^Na MRI studies [37]. The choice of the optimal flip angle was made according to SNR measurements with different FAs; the higher SNR was found at FA = 30°. To confirm the experimental finding, we calculated the Ernst angle that maximizes the MR signal for a given T1 and TR: considering T1 to be about 60 ms [46] and a chosen TR = 5 ms, the resulting Ernst angle is 24°, which is very similar to the experimental value. 

In this paper, the CoV was used as an index of intra- and inter-day measurement repeatability [41]. Using the optimized acquisition protocols, a high repeatability (CoV < 20%) of the measurement for intra- and inter-day MRI sessions was found for all the vials with ^23^Na solutions at high concentrations (38.5, 77 mM in Table 3). The CoVs were generally lower for the higher concentration values and larger vials, suggesting higher measurement repeatability. For the smaller vials and the lower concentrations (9.625, 19.25 mM) assessing the repeatability of the measurements is critical because of the SNR limitations. The SNR obtained could be improved with signal averaging, although at the cost of increased scan time. Envisaging the translation of the proposed method to human studies, we should consider that MR acquisitions with a long scan time can result in poor patient comfort and, in the worst case, induce movement artifacts. The acquisitions performed in this study had a total scan time of about 15 min, which could be a good compromise between suitable image quality and scan time. 

In this work, we took our experimental setup to the limit of sensitivity to determine the detection limit of the method. As in vivo TSCs are in the same order of magnitude as the high concentration vials [27], this suggests a good reliability of the acquisitions presented here. As previously described, some criticalities were found for imaging of the lowest concentration values in the ^23^Na phantom. Since these concentration values should be lower than the physiological values, we expect the SNR limitation to be less critical when translating the proposed approach to in vivo studies.

The measurements reported in this study were performed with an acquisition sequence that can be further optimized for human studies in different tissues/organs, suggesting the potential of our approach for clinical application.

The obtained results suggest the reproducibility of our protocol and confirm the suitability of our method for performing longitudinal ^23^Na MRI QA and QC studies.

## 5. Conclusions

Sodium tissue imaging and quantification represents a new challenge for observing physiological and pathophysiological processes from a different perspective. This may potentiate the assessment of diagnostic and prognostic stratification in patients with several acute and chronic organ diseases, therapeutic decisions, as well as environmental factors, i.e., diet and exercise, for primary and secondary prevention.

Because of the intrinsic low sensitivity of ^23^Na MRI, testing the repeatability and reproducibility of the acquisition process is critical to ensure reliable data comparison and proper understanding of the results.

In this paper, we describe an experimental setup and methodology for performing ^23^Na MRI studies in phantom, which could be suitable for human studies, and we investigate its reproducibility and repeatability over a medium time period. 

We believe that the proposed method could be a promising starting point for the development of ^23^Na MRI human studies performed under different physio-pathological conditions in a clinical setting.

Despite the fact that further work is needed to improve the performance of the technology, our results support the reliability of ^23^Na MR imaging as a powerful semi-quantitative tool with an interesting applicability for in vivo non-invasive human studies.

## Figures and Tables

**Table 1:** SNR measurement for FA = 15° and FA = 30°.

	SNR 1 Day	SNR 2 Day	SNR 3 Day
FA = 15°	70	61	60
FA = 30°	79	65	64

**Table 2:** The ^23^Na concentration values [mM] estimated for each vial (the results are presented as mean ± standard deviation).

Vial Size	Nominal Concentration [mM]	Estimated Concentration [mM] FA 15°	Estimated Concentration [mM] FA 30°
Large	77	61.5 ± 2.4	68.4 ± 2.1
Large	38.5	52.5 ± 0.6	46.4 ± 1.0
Large	19.25	25.6 ± 2.2	23.6 ± 1.7
Large	9.625	10.5 ± 1.4	13.5 ± 1.1
Medium	77	55.5 ± 1.4	67.1 ± 2.9
Medium	38.5	32.6 ± 3.5	31.0 ± 0.7
Medium	19.25	19.5 ± 1.4	15.3 ± 1.9
Medium	9.625	2.0 ± 2.0	0.8 ± 2.0
Small	77	39.2 ± 3.5	45.7 ± 2.4
Small	38.5	17.5 ± 2.3	17.4 ± 2.3
Small	19.25	4.6 ± 5.1	2.2 ± 2.0
Small	9.625	−3.1 ± 1.2	−3.1 ± 2.0
Small (Central)	154	96.9 ± 6.3	112.8 ± 6.6

**Table 3:** Intra- and inter-day repeatability of the sodium concentration values for the different vials of the phantom, evaluated through the CoV. The vials are identified by a label indicating the size (L = large; M = medium; S = small) and the concentration value.

	INTRA-DAY Repeatability		INTER-DAY Repeatability	
Vial	CV(%) FA 15°	CV(%) FA 30°	CV(%) FA 15°	CV(%) FA 30°
L_77	1.06	1.69	4.98	3.96
L_38.5	1.48	2.27	1.44	2.27
L_19.3	3.60	4.90	6.34	9.72
L_9.6	11.89	5.63	13.56	4.85
M_77	0.88	2.78	3.30	3.77
M_38.5	6.56	2.96	12.97	0.62
M_19.3	7.92	17.57	2.66	6.68
M_9.6	>50.0	>50.0	>50.0	>50.0
S_77	6.27	5.70	4.04	3.61
S_38.5	9.62	4.37	9.20	18.52
S_19.3	>50.0	>50.0	>50.0	>50.0
S_9.6	11.92	>50.0	44.50	>50.0
Centre_154	0.57	4.76	9.19	4.96

## Data Availability

Data are contained within the article.
